# Supplementary figures and images for: The impact of a demand-side sanitation and hygiene promotion intervention on sustained behavior change and health in Amhara, Ethiopia: A cluster-randomized trial
Source: PLOS Glob Public Health. 2022 Jan 7;2(1):e0000056. doi: 10.1371/journal.pgph.0000056 (PMC10021625; doi:10.1371/journal.pgph.0000056)

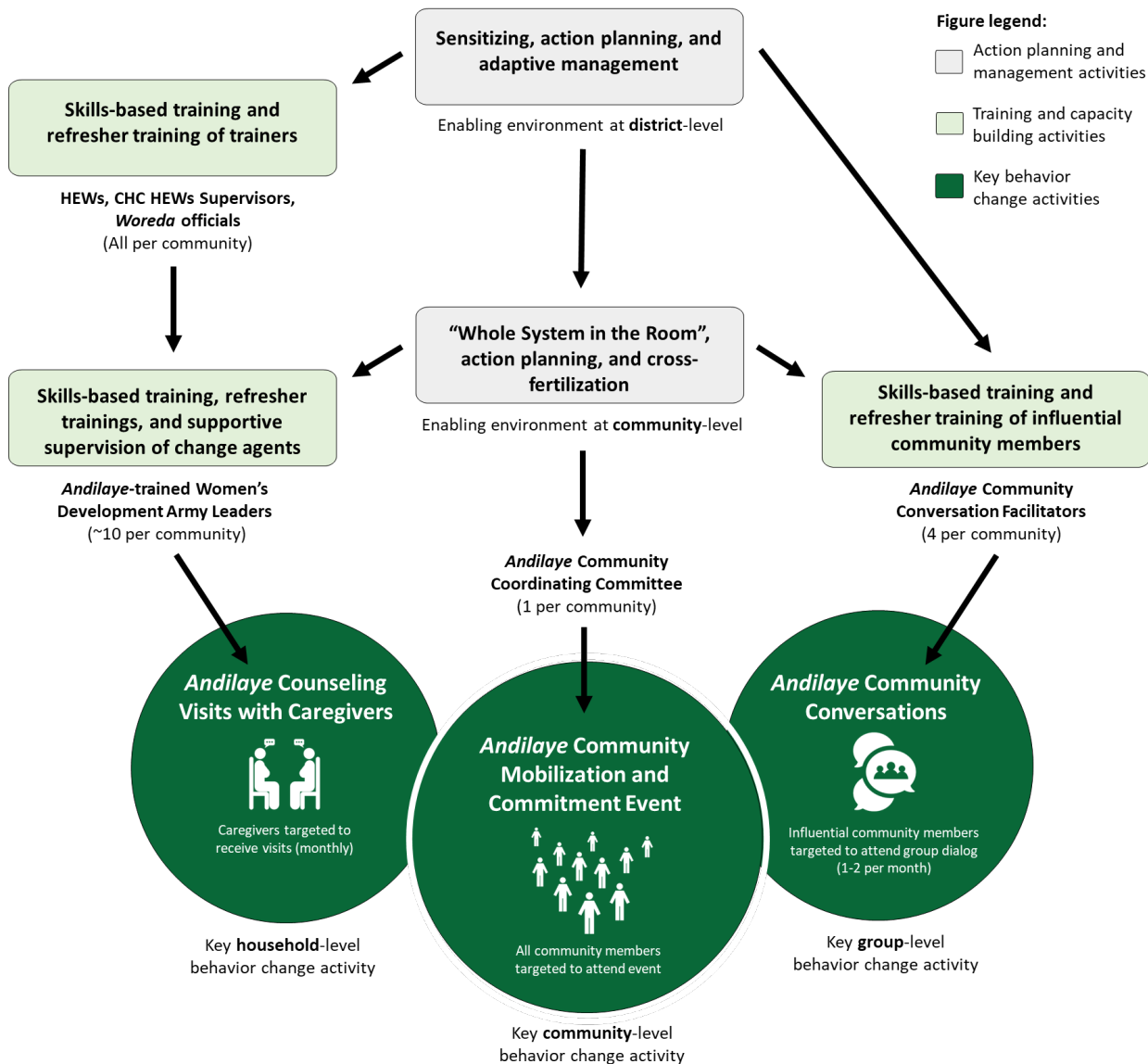

**Supplemental Figure 1.** Diagram summarizing the *Andilaye* intervention

Supplement: S1 Fig — (PDF) [file pgph.0000056.s001.pdf]
