# Supplementary material for: The impact of a demand-side sanitation and hygiene promotion intervention on sustained behavior change and health in Amhara, Ethiopia: A cluster-randomized trial
Source: PLOS Glob Public Health. 2022 Jan 7;2(1):e0000056. doi: 10.1371/journal.pgph.0000056 (PMC10021625; doi:10.1371/journal.pgph.0000056)
Supplement: S1 Table — (PDF) [file pgph.0000056.s002.pdf]

Supplemental Table 1. Summary of the *Andilaye* intervention activities and dates of delivery

Action planning and management	Activity aim	Level (type)	Dates of delivery [a]
(1) Sensitizing and action planning workshop	To orient key stakeholders to the *Andilaye* intervention and engage them in intervention action planning so as to generate buy-in and foster an enabling environment in which the intervention can be implemented.	District (catalyzing)	September to October 2017
(2) Whole system in the room and action planning	To engage key community stakeholders, orient them to the *Andilaye* intervention, and facilitate their involvement in intervention action planning. This participatory approach aims to generate buy-in and foster an enabling environment (i.e., social opportunity) in which the *Andilaye* intervention can be supported and effectively implemented for a "*strong, caring, healthy community*."	Community (catalyzing)	January to March 2018
(3) Adaptive management workshop	To leverage monitoring data to facilitate evidence-based, controlled, and documented operational-specific modifications during critical program moments (i.e., "change gates"). To improve intervention outcomes and resource management by learning from monitored program outcomes.	District (maintenance)	February to March 2019 [c]
(4) Cross-fertilization visits	To provide an opportunity to share experiences across different intervention communities – to address common implementation bottlenecks, propose solutions, and share perspectives on preliminary behavior change and health outcomes.	Community (maintenance)	February to March 2019 [b]
**Training and capacity building**	**Activity aim**	**Level (type)**	**Dates of delivery [a]**
(1) Skills-based training of the trainers for HEWs, CHC HEWs Supervisors, *Woreda* officials	To provide skills-based training to Health Extension Workers (HEWs), Cluster Health Center (CHC) HEWs Supervisors, and *Woreda* officials on household-level intervention activities, supportive supervision, and on-the-job-training so HEWs can, in turn, effectively train Women's Development Army Leaders (WDALs) on the implementation of household-level activities and provide supportive supervision.	District (catalyzing)	December 2017 to January 2018
(2) Skills-based training of WDALs	To provide skills-based training to WDALs on household-level intervention activities, as detailed in the training of the trainers for HEWs, CHC HEWs Supervisors, and *Woreda* officials.	Community (catalyzing)	January to February 2018
(3) Training of community conversation facilitators	To provide comprehensive facilitator training to selected *gott* and *kebele* stakeholders on the 'community conversations' group-level intervention activity.	District (catalyzing)	August to October 2018 [b]
(4) Skills-based refresher training of the trainers for HEWs, CHC HEWs Supervisors, *Woreda* officials	To reinforce previously acquired knowledge and skills and address trainer/facilitator turnover. Prior experience indicates that such trainings serve to sustain actor motivation and further strengthen capacity.	District (maintenance)	November 2018

(5) Skills-based review meetings and refresher trainings for WDALs (round 1)	To reinforce previously acquired knowledge and skills, address WDAL turnover, and review successes and address challenges faced in implementing counseling visits with caregivers. Prior experience indicates that such trainings serve to sustain actor motivation and further strengthen capacity.	Community (maintenance)	November to December 2018
(6) Skills-based review meetings and refresher trainings for WDALs (round 2)	To reinforce previously acquired knowledge and skills, address WDAL turnover, and review successes and address challenges faced in implementing counseling visits with caregivers. Prior experience indicates that such trainings serve to sustain actor motivation and further strengthen capacity.	Community (maintenance)	February to March 2019
(7) Skills-based refresher training of community conversation facilitators	To reinforce previously acquired knowledge and skills and address trainer/facilitator turnover. Prior experience indicates that such trainings serve to sustain actor motivation and further strengthen capacity.	District (maintenance)	February to April 2019 [b]
**Key behavior change activities**	**Activity aim**	**Level (type)**	**Dates of delivery [a]**
(1a) Counseling visits with caregivers	To provide personalized counseling to caregivers to equip them with the knowledge, skills, and motivation necessary to adopt improved WASH practices. To foster action capacity, self-efficacy, and barrier planning so caregivers maintain the improved WASH practices.	Household (catalyzing)	February 2018 to May 2019 – As dictated by HEWs and WDALs after trainings
(1b) Follow-up barrier planning counseling visits with caregivers	To provide continuous follow-up to households such that the house graduates from counseling related to initial adoption of improved practices to counseling related to behavioral maintenance skills. These visits will progressively focus more and more on specific barrier identification and planning skills so the caregiver can maintain his/her improved WASH practices, especially as personal setbacks, systemic shocks, and other obstacles arise.	Household (maintenance)	As dictated by household progress
(2) Community mobilization and commitment event	To improve action knowledge, barrier identification and planning, and attitudes regarding targeted NTD-preventive WASH behaviors through a form of contextually appropriate and interactive edutainment. To initiate the process of shifting social norms through community-generated and managed by-laws and sanctions and public commitment thereof.	Community (catalyzing)	March to April 2018
(3a) Community conversations	To change factual beliefs and attitudes, enhance action knowledge, improve perceptions of capability, identify and make plans to overcome barriers, and shift social norms regarding targeted behaviors through community group dialogue. To carry out demonstrations that address key factors associated with both breaking away from unimproved practices and adopting improved sanitation and hygiene practices.	Group (catalyzing)	October 2018 to May 2019 – As dictated by facilitators after trainings

(3b) Follow-up community conversations	To generate community-level dialogue regarding nuanced issues associated with maintenance of improved practices and barriers thereof through a follow-up round of community group dialog. To carry-out demonstrations related to behavioral maintenance issues.	Group (maintenance)	As dictated by group progress

[a] Dates reported reflect the overall range in which activities were delivered among all *woredas* and intervention *kebeles*. [b] Considerable delays were faced in scheduling with *woreda* officials of Bahir Dar Zuria resulting in a wider range of delivery dates.
