# Supplementary material for: The impact of a demand-side sanitation and hygiene promotion intervention on sustained behavior change and health in Amhara, Ethiopia: A cluster-randomized trial
Source: PLOS Glob Public Health. 2022 Jan 7;2(1):e0000056. doi: 10.1371/journal.pgph.0000056 (PMC10021625; doi:10.1371/journal.pgph.0000056)
Supplement: S2 Table — (PDF) [file pgph.0000056.s003.pdf]

Supplemental Table 2. Alignment of relevant roles and responsibilities of the Ethiopian Health Extension Programme (HEP) and *Andilaye* Trial

Stakeholder	Relevant HEP roles and responsibilities [a]	Alignment with *Andilaye* Trial
**The Federal Ministry of Health**	**Design:** Determine overall program concept, standards, and implementation guidelines and provide communication tools and materials	✓ Engaged during formative research and intervention design
	**Implementation (National-level):** Mobilize national and international resources	✗ National implementation and reporting roles and responsibilities were not included as rolling out a government-sponsored program in a select number of areas was both politically and logistically infeasible
	**Reporting:** Establish the health management information system	
**The Regional Health Bureaus/Zonal Health Departments**	**Design:** Adapt implementation guidelines to local conditions and communication tools; and adapt materials into local languages and distributes them to woredas	✓ Engaged during formative research and intervention design
	**Implementation (Regional/Zonal-level):** Provide technical and administrative support to *Woreda* Health Offices	✗ Regional/Zonal implementation and reporting roles and responsibilities were performed by the Ethiopian-based study team
	**Reporting:** Obtain reports from *Woreda* Health Offices and provide information to the Ministry of Health	
**The *Woreda* Health Office**	**Design:** Adapt communication tools and materials	✓ Engaged during formative research and intervention design
	**Implementation (District-level):** Provide technical, administrative, and financial support to health centers and health posts; and plan and provide in-service training to HEWs and *Woreda* Health Office staff	✓ While financial and overall technical support was provided by the *Andilaye* team, the *Woreda* Health Office was designed as the key stakeholder in implementing all district-level activities
	**Supportive supervision:** Provide supportive supervision of HEWs and the overall management of health centers and health posts	✓ Supportive supervision and on-the-job-training of HEWs were designed to be completed by HEWs Supervisors via an *Andilaye*-specific checklist
	**Reporting:** Obtain reports from health posts and health centers and provide information to Regional Health Bureaus or Zonal Health Departments	✓ While detailed process data was collected by the Ethiopian-based study team, program monitoring mechanisms were included in the design and district-level action planning tool
**Health Extension Workers (HEWs)**	**Implementation (Community-level):** Manage operations of health posts; and identify, train, and collaborate with WDALs teams	✓ While overall technical support was provided by the Ethiopian-based study team, the HEW was designed as the key stakeholder in implementing all community-level activities and engaging additional community change agents for group-level and household-level activities
	**Implementation (Group-level):** Communicate health messages by involving the community social	✓ Over-extension of HEWs was addressed through the engagement of additional community change agents for group-level

	networks, associations, religious institutions, and government structures		activities (i.e., community conversation facilitators) and household-level activities (i.e., WDALs)
	**Implementation (Household-level):** Conduct home visits and outreach services to promote preventive actions; and prioritize households with low performance in implementing the package and support them in all the Health Extension Packages that are relevant to them		
	**Supportive supervision:** Provide supportive supervision and evaluation of WDAL teams; and conduct biweekly meetings to evaluate the performance of the development teams	✓	Supportive supervision and on-the-job-training of WDALs was designed to be completed by HEWs via an *Andilaye*-specific checklist
	**Reporting:** Provide reports to *Woreda* Health Offices	✓	While detailed process data was collected by the Ethiopian-based study team, program monitoring mechanisms were included in the design and community-level action planning tool
**Women's Development Army Leaders (WDALs)**	**Implementation (Community, group, and household-level):** Volunteers not institutionalized into the health system; they are regarded as community representatives and reflect government efforts to devolve responsibilities for health to individuals and local communities by mobilizing the population and supporting HEWs	✓	In addition to supporting community and group-level activities, WDALs were trained to provide inter-personal counseling with caregivers by conducting *Andilaye* household counseling visits with each household in her catchment area

[a] Workie, N.W. and R., Gandham NV, 2013. Ethiopia - The health extension program in Ethiopia, Universal Health Coverage (UNICO) studies series. World Bank, Washington, DC.
