# Supplementary material for: The impact of a demand-side sanitation and hygiene promotion intervention on sustained behavior change and health in Amhara, Ethiopia: A cluster-randomized trial
Source: PLOS Glob Public Health. 2022 Jan 7;2(1):e0000056. doi: 10.1371/journal.pgph.0000056 (PMC10021625; doi:10.1371/journal.pgph.0000056)
Supplement: S3 Table — (PDF) [file pgph.0000056.s004.pdf]

Supplemental Table 3. Summary of process data for the *Andilaye* Trial

Supplemental Table 3a. Process data for *Andilaye* action planning and management intervention activities

**(1) Sensitizing and action planning workshop**					
District-level catalyzing activity		Bahir Dar Zuria	Fogera	Farta	Overall
Dose delivered	Proportion of districts with activity implemented (September-October 2017)	1/1 (100%)	1/1 (100%)	1/1 (100%)	3/3 (100%)
	Proportion of districts with activity objectives completed as planned (according to activity checklist)	1/1 (100%)	1/1 (100%)	1/1 (100%)	3/3 (100%)
	Length of activity (one day workshop)	8 hours	7.5 hours	4.75 hours [a]	6.75 hours
Participation	Total number of participants	19 [b]	12	16	47
	Proportion of districts with Amhara Regional Health Bureau OR COWASH representative in attendance	1/1 (100%)	1/1(100%)	0/1 (0%)	2/3 (66%)
	Proportion of districts with *Woreda* Health Office representative AND Hygiene and Sanitation Officer in attendance	1/1 (100%)	1/1 (100%)	1/1 (100%)	3/3 (100%)
	Proportion of intervention *kebeles* with administrator in attendance	7/8 (88%) [c]	6/6 (100%)	11/11 (100%)	24/25 (96%)
Dose received	Proportion of districts with endorsed action plan for the completion of district-level activities and the ‘ Whole system in the room and action planning’ community-level activity and accompanying supportive supervision	1/1 (100%)	1/1 (100%)	1/1 (100%)	3/3 (100%)
	Proportion of participants that endorsed district-level action plan	15/19 (79%)	12/12 (100%)	15/16 (94%)	42/47 (89%) [d]
Context	[a] Participants had another *woreda*-level governmental workers’ meeting with the *woreda* administrator and asked to finish this workshop early. The action plan was supposed to be first completed in two groups and then each group presenting to all participants to finalize. To accommodate the other meeting, the participants completed the action plan as one group and finalized.<br>[b] The workshop completed in Bahir Dar City (the regional capital city) was the one with the highest stakeholder turnout, including regional level government and non-government organizations stakeholders.<br>[c] One Bahir Dar Zuria *kebele* administrator from a non-study *kebele* (Wonjeta) was invited by mistake instead the *kebele* administrator from an intervention *kebele* (Wondata). Similarity of names created the mismatch while extending invitations.<br>[d] Some workshop participants (mainly regional representatives and *woreda* administrators) did not attend the afternoon session of the workshop; this impacted the number stakeholders endorsing action plans.				
**(2) Whole system in the room and action planning**					
Community-level catalyzing activity		Bahir Dar Zuria	Fogera	Farta	Overall
Dose delivered	Proportion of intervention *kebeles* with activity implemented (January-March 2018)	8/8 (100%)	6/6 (100%)	11/11 (100%)	25/25 (100%)
	Proportion of intervention *kebeles* with activity objectives completed as planned (according to activity checklist)	8/8 (100%)	6/6 (100%)	11/11 (100%)	25/25 (100%) [a]
	Average length of activity (partial day workshop)	1.75 hours	2 hours	1.33 hours	1.75 hours
Participation	Total number of participants	64	42	117	223

	Proportion of intervention *kebeles* with administrator acting as activity facilitator	7/8 (88%)	6/6 (100%)	11/11 (100%)	24/25 (96%)
	Proportion of intervention *kebeles* with *Woreda* Health Office representatives acting as activity co-facilitator	1/8 (13%)	0/6 (0%)	1/11 (9%)	2/25 (8%)
	Proportion of intervention *kebeles* with manager in attendance	7/8 (88%)	6/6 (100%)	9/11 (82%)	22/25 (88%)
	Proportion of intervention *kebeles* with at least one Health Extension Worker (HEW) in attendance	8/8 (100%)	6/6 (100%)	11/11 (100%)	25/25 (100%)
	Proportion of intervention *kebeles* with at least one Agriculture Extension Worker (AEW) in attendance	7/8 (88%)	6/6 (100%)	10/11 (91%)	23/25 (92%)
	Proportion of intervention *kebeles* with at least one school director in attendance	5/8 (63%)	5/6 (83%)	5/11 (45%)	15/25 (60%)
	Proportion of intervention *kebeles* with at least one Women's Development Army Leader (WDAL) in attendance	4/8 (50%)	1/6 (17%)	7/11 (64%)	12/25 (48%)
	Proportion of intervention *kebeles* with at least one religious leader in attendance	7/8 (88%)	4/6 (67%)	10/11 (91%)	21/25 (84%)
	Proportion of intervention *kebeles* with at least one influential elder or other influential people from the *gott*	8/8 (100%)	4/6 (67%)	11/11 (100%)	22/25 (88%)
Dose received	Proportion of intervention *kebeles* with *Andilaye* community conversation facilitators identified in the action plan	8/8 (100%)	6/6 (100%)	11/11 (100%)	25/25 (100%)
	Proportion of intervention *kebeles* with a coordinating committee for the 'Community mobilization and commitment event' identified in the action plan	8/8 (100%)	6/6 (100%)	11/11 (100%)	25/25 (100%)
	Proportion of intervention *kebeles* with a master of ceremony for the 'Community mobilization and commitment event' identified in the action plan	8/8 (100%)	6/6 (100%)	11/11 (100%)	25/25 (100%)
Context	[a] The objectives were completed as planned, however, the expectation of payment (per diem) associated with the activity was an issue and in some cases a clear disappointment for participants. Although this was a one to two hour activity and government policies were followed, participants (a composition of community members and salaried government workers) seem to expect some kind of per diem and when they found out that there is no per diem (at the end of the activity), some participants expressed their disappointment.				
**(3) Adaptive management workshop and cross-fertilization visits**					
District and community-level maintenance activities		Bahir Dar Zuria	Fogera	Farta	Overall
Dose delivered	Proportion of districts with activity implemented (Feb-Mar 2019)	1/1 (100%)	1/1 (100%)	1/1 (100%)	3/3 (100%) [a]
	Proportion of districts with activity objectives completed as planned (according to activity checklist)	1/1 (100%)	1/1 (100%)	1/1 (100%)	3/3 (100%)
	Length of activity each day (two day workshop)	8 hours	8 hours	8 hours	8 hours
Participation	Total number of participants	25	18	28	71
	Proportion of districts with *Woreda* Health Office Head, Hygiene and Sanitation Officer, AND HEW Program Officer in attendance	1/1 (100%)	1/1 (100%)	1/1 (100%)	3/3 (100%)
	Proportion of intervention *kebele* Cluster Health Centers (CHC) Heads AND CHC HEWs Supervisors in attendance	7/7 (100%)	4/4 (100%)	7/7 (100%)	18/18 (100%)

	Proportion of intervention *kebeles* with administrator in attendance	8/8 (100%)	6/6 (100%)	11/11 (100%)	25/25 (100%)
Dose received	Proportion of districts that identified implementation challenges and developed action plan for ways forward for the completion of district, community, group, and household-level activities	1/1 (100%)	1/1 (100%)	1/1 (100%)	3/3 (100%) [b]
Context	[a] Cross fertilization visits were conducted on the second day of the workshop where all participants visited 'better performing' intervention *kebeles* (as selected on during the first day of the adaptive management workshop). During the visit, participants observed WDALs performing household visits and facilitators conducting community conversations. After observing these activities, the participants came together to further discuss ways forward to overcome implementation challenges in their respective communities.<br>[b] The overall lack of ownership of project activities and existing gaps in implementing the Health Extension Program (HEP) (e.g., supportive supervision and WDAL structure) were major implementation challenge discussed in all districts.				

Supplemental Table 3b. Process data for *Andilaye* training and capacity building intervention activities

**(1) Skills-based training of the trainers for HEWs, CHC HEWs Supervisors, *Woreda* officials**					
District-level catalyzing activity		Bahir Dar Zuria	Fogera	Farta	Overall
Dose delivered	Proportion of districts with activity implemented (December 2017-January 2018)	1/1 (100%)	1/1 (100%)	1/1 (100%)	3/3 (100%)
	Proportion of districts with activity objectives completed as planned (according to activity checklist)	1/1 (100%)	1/1 (100%)	1/1 (100%)	3/3 (100%)
	Length of activity each day (two day training)	10 hours	10 hours	10 hours	10 hours
Participation	Total number of participants	38	27	44 [a]	108
	Proportion of districts with Woreda Health Office representative AND Hygiene and Sanitation Officer in attendance	1/1 (100%)	1/1 (100%)	1/1 (100%)	3/3 (100%) [b]
	Proportion of intervention kebele CHC HEWs Supervisors trained	7/7 (100%)	4/4 (100%)	7/7 (100%)	18/18 (100%)
	Proportion of intervention kebele HEWs trained	21/21 (100%)	15/15 (100%)	20/21 (95%)	56/57 (98%) [b]
	Proportion of intervention kebeles with at least one HEW trained	8/8 (100%)	6/6 (100%)	11/11 (100%)	25/25 (100%)
Dose received	Proportion of intervention kebeles with endorsed action plan for the completion of household-level activities and accompanying supportive supervision	8/8 (100%)	6/6 (100%)	11/11 (100%)	25/25 (100%)
Context	[a] Two training sessions were conducted in Farta given the high number of intervention kebeles and target participants.<br>[b] A "mop up" training was conducted in Bahir Dar City after the district-level trainings to address 10 HEWs (6 from Bahir Dar Zuria, 2 from Fogera, and 2 from Farta) and Bahir Dar Zuria Woreda Health Office representative and Hygiene and Sanitation Officer that were absent during the original trainings.				
**(2) Skills-based training of WDALs**					
Community-level catalyzing activity		Bahir Dar Zuria	Fogera	Farta	Overall
Dose delivered	Proportion of intervention kebeles with activity implemented (January-February 2018)	8/8 (100%)	6/6 (100%)	11/11 (100%)	25/25 (100%)
	Proportion of intervention kebeles with activity objectives completed as planned (according to activity checklist)	8/8 (100%)	6/6 (100%)	11/11 (100%)	25/25 (100%)
	Average length of activity each day (two day training)	4.75 hours	4.0 hours	7 hours	6 hours
Participation	Proportion of intervention kebeles with HEWs acting as activity training facilitator	8/8 (100%)	6/6 (100%)	11/11 (100%)	25/25 (100%)
	Proportion of intervention kebeles with CHC HEWs Supervisors acting as activity training co-facilitator	6/8 (75%)	6/6 (100%)	8/11 (73%)	20/25 (80%) [a]
	Proportion of WDALs from intervention kebeles in attendance during the training (8-10 per kebele)	72/73 (99%)	54/56 (96%)	107/110 (97%)	233/239 (97%)
	Proportion of intervention kebeles with Andilaye trained WDALs	8/8 (100%)	6/6 (100%)	11/11 (100%)	25/25 (100%)
Dose received	Proportion of intervention kebeles with HEWs reporting that they have received Andilaye supportive supervision from CHC HEWs Supervisors after training [during quarterly monitoring surveys in July 2018]	0/8 (0%)	2/6 (33%)	0/11 (0%)	2/25 (8%) [b]

	Proportion of randomly sampled WDALs reporting that they have received Andilaye supportive supervision from HEWs after training [during quarterly monitoring surveys in July 2018]	12/29 (41%)	4/23 (17%)	21/42 (50%)	37/94 (39%) [b]
Context	[a] Training of WDALs was planned to primarily be facilitated by HEWs with help from CHC HEWs Supervisors, but the involvement of CHC HEWs Supervisors was minimal in some kebeles. Thus, the Ethiopian-based study team assisted trainers and trained WDALs together with HEWs.<br>[b] It was recommended that CHC HEWs Supervisors perform supportive supervision on a routine basis (at least once per month for each HEW), in accordance with the woreda's Andilaye action planning document. Similarly, it was recommended that HEWs perform supportive supervision with each WDAL in her catchment area every month, in accordance with the HEW's Andilaye action planning document. When conducting supervisory visits, HEWs were trained to use the supportive supervision and on-the-job checklist. However, quarterly monitoring surveys conducted 5-6 months after the training of WDALs (July 2018) indicated that few HEWs have received supportive supervision and on-the-job training from CHC HEW Supervisors and that few WDALs have received supportive supervision and on-the-job training from HEWs. The review of the importance of providing supportive supervision to both HEWs and WDALs was prioritized during 'Skills-based refresher training of the trainers for HEWs, CHC HEWs Supervisors, Woreda officials' (November 2018) and during 'Skills-based review meetings and refresher trainings for WDALs (round 1)' (November-December 2018).				
**(3) Training of community conversation facilitators**					
District-level catalyzing activity		Bahir Dar Zuria	Fogera	Farta	Overall
Dose delivered	Proportion of districts with activity implemented (August-October 2018)	1/1 (100%)	1/1 (100%)	1/1 (100%)	3/3 (100%)
	Proportion of districts with activity objectives completed as planned (according to activity checklist)	1/1 (100%)	1/1 (100%)	1/1 (100%)	3/3 (100%)
	Length of activity each day (two day training)	8.33 hours	8.75 hours	7.75 hours	8.25 hours
Participation	Total number of participants	34 [a]	26	45 [a]	105
	Proportion of districts with Woreda Health Office representative AND Hygiene and Sanitation Officer in attendance	1/1 (100%)	0/1 (0%)	1/1 (100%)	2/3 (66%)
	Proportion of Andilaye community conversation facilitators in attendance	32/32 (100%)	24/24 (100%)	43/44 (98%)	99/100 (99%)
	Proportion of intervention kebeles with at least at least one facilitator per pair of facilitators trained on Andilaye community conversations	8/8 (100%)	6/6 (100%)	11/11 (100%)	25/25 (100%)
Dose received	Proportion of intervention kebeles with facilitators that endorsed action plan for the completion of group-level activities	8/8 (100%)	6/6 (100%)	11/11 (100%)	25/25 (100%) [b]
Context	[a] Two training sessions were conducted in Bahir Dar Zuria and Farta given the high number of intervention kebeles and target participants.<br>[b] During action planning, it was nearly unanimous that refreshments would need to be provided in order to motivate community members to attend community conversations, as planned. While this was not originally budgeted, each intervention kebele was provided coffee and flour for bread to accommodate a coffee ceremony during the community conversations.				
**(4) Skills-based refresher training of the trainers for HEWs, CHC HEWs Supervisors, *Woreda* officials**					
District-level maintenance activity		Bahir Dar Zuria	Fogera	Farta	Overall
Dose delivered	Proportion of districts with activity implemented (November 2018)	1/1 (100%)	1/1 (100%)	1/1 (100%)	3/3 (100%)
	Proportion of districts with activity objectives completed as planned (according to activity checklist)	1/1 (100%)	1/1 (100%)	1/1 (100%)	3/3 (100%)

	Length of activity each day (two day training)	8 hours	8 hours	8 hours	8 hours
Participation	Total number of participants	29	26	46 [a]	101
	Proportion of study districts with Woreda Health Office representative AND Hygiene and Sanitation Officer in attendance during training	0/1 (0%)	1/1 (100%)	1/1 (100%)	2/3 (66%)
	Proportion of intervention kebele CHC HEWs Supervisors trained	7/7 (100%)	4/4 (100%)	5/7 (88%)	16/18 (89%)
	Proportion of intervention kebele HEWs trained	16/21 (100%)	13/15 (88%)	25/25 (100%)	54/61 (89%)
	Proportion of intervention kebeles with at least one HEW trained	8/8 (100%)	6/6 (100%)	11/11 (100%)	25/25 (100%)
Dose received	Proportion of intervention kebeles that identified implementation challenges and developed action plan for ways forward for the completion of household-level activities and accompanying supportive supervision	8/8 (100%)	6/6 (100%)	11/11 (100%)	25/25 (100%)
Context	[a] Two training sessions were conducted in Farta given the high number of intervention kebeles and target participants.				
**(5) Skills-based review meeting and refresher training for WDALs (round 1)**					
Community-level maintenance activity		Bahir Dar Zuria	Fogera	Farta	Overall
Dose delivered	Proportion of intervention kebeles with activity implemented (November-December 2018)	8/8 (100%)	6/6 (100%)	11/11 (100%)	25/25 (100%)
	Proportion of intervention kebeles with activity objectives completed as planned (according to activity checklist)	8/8 (100%)	6/6 (100%)	11/11 (100%)	25/25 (100%)
	Average length of activity (partial day training)	2.75 hours	3.0 hours	3.25 hours	3 hours
Participation	Proportion of intervention kebeles with HEWs acting as activity training facilitator	8/8 (100%)	6/6 (100%)	11/11 (100%)	25/25 (100%)
	Proportion of intervention kebeles with CHC HEWs Supervisors acting as activity training co-facilitator	7/8 (88%)	6/6 (100%)	10/11 (91%)	23/25 (92%) [a]
	Proportion of WDALs from intervention kebeles in attendance during the training (8-10 per kebele)	70/74 (95%)	62/62 (100%)	104/107 (97%)	236/243 (97%) [b]
	Proportion of intervention kebeles with Andilaye trained WDALs	8/8 (100%)	6/6 (100%)	11/11 (100%)	25/25 (100%)
Dose received	Proportion of intervention kebeles with HEWs reporting that they have received Andilaye supportive supervision from CHC HEWs Supervisors after training [during quarterly monitoring surveys in December 2018]	0/8 (0%)	0/6 (0%)	1/11 (9%)	1/25 (4%) [c]
	Proportion of randomly sampled WDALs reporting that they have received Andilaye supportive supervision from HEWs after training [during quarterly monitoring surveys in December 2018]	15/29 (52%)	5/24 (21%)	18/43 (42%)	38/96 (40%) [c]
Context	[a] Training of WDALs was planned to primarily be facilitated by HEWs with help from CHC HEWs Supervisors, but the involvement of CHC HEWs Supervisors was minimal in some kebeles. Thus, the Ethiopian-based study team assisted trainers and trained WDALs together with HEWs.<br>[b] Some kebeles added/removed small numbers of WDALs responsible for conducting Andilaye counseling visits with caregivers.<br>[c] It was recommended that CHC HEWs Supervisors perform supportive supervision on a routine basis (at least once per month for each HEW), in accordance with the woreda's Andilaye action planning document. Similarly, it was recommended that HEWs perform supportive				

	supervision with each WDAL in her catchment area every month, in accordance with the HEW's Andilaye action planning document. When conducting supervisory visits, HEWs were trained to use the supportive supervision and on-the-job checklist. However, quarterly monitoring surveys conducted 1-2 months after the review meeting and refresher training of WDALs (December 2018) indicated that few HEWs have received supportive supervision and on-the-job training from CHC HEW Supervisors and that few WDALs have received supportive supervision and on-the-job training from HEWs. The review of the importance of providing supportive supervision to both HEWs and WDALs was prioritized during 'Adaptive management workshop' (February-March 2019) and during 'Skills-based review meetings and refresher trainings for WDALs (round 2)' (February-March 2019).				
**(6) Skills-based review meeting and refresher training for WDALs (round 2)**					
Community-level maintenance activity		Bahir Dar Zuria	Fogera	Farta	Overall
Dose delivered	Proportion of intervention kebeles with activity implemented (February-March 2019)	8/8 (100%)	6/6 (100%)	11/11 (100%)	25/25 (100%)
	Proportion of intervention kebeles with activity objectives completed as planned (according to activity checklist)	8/8 (100%)	6/6 (100%)	11/11 (100%)	25/25 (100%)
	Average length of activity (one day training)	2.5 hours	2.5 hours	2.66 hours	2.5 hours
Participation	Proportion of intervention kebeles with HEWs acting as activity training facilitator	8/8 (100%)	6/6 (100%)	11/11 (100%)	25/25 (100%)
	Proportion of intervention kebeles with CHC HEWs Supervisors acting as activity training co-facilitator	2/8 (25%)	3/6 (50%)	4/11 (36%)	9/25 (36%) [a]
	Proportion of WDALs from intervention kebeles in attendance during the training (8-10 per kebele)	71/78 (91%)	53/61 (87%)	103/107 (96%)	227/246 (92%) [b]
	Proportion of intervention kebeles with Andilaye trained WDALs	8/8 (100%)	6/6 (100%)	11/11 (100%)	25/25 (100%)
Dose received	Proportion of intervention kebeles with HEWs reporting that they have received Andilaye supportive supervision from CHC HEWs Supervisors after training [during endline surveys in May 2019]	0/8 (0%)	3/6 (50%)	2/11 (18%)	5/25 (20%) [c]
	Proportion of randomly sampled WDALs reporting that they have received Andilaye supportive supervision from HEWs after training [during endline surveys in May 2019]	9/32 (28%)	5/24 (21%)	29/44 (66%)	43/100 (43%) [c]
Context	[a] Training of WDALs was planned to primarily be facilitated by HEWs with help from CHC HEWs Supervisors, but the involvement of CHC HEWs Supervisors was minimal in some kebeles. Thus, the Andilaye team assisted trainers and trained WDALs together with HEWs.<br>[b] Some kebeles added/removed small numbers of WDALs responsible for conducting Andilaye counseling visits with caregivers.<br>[c] It was recommended that CHC HEWs Supervisors perform supportive supervision on a routine basis (at least once per month for each HEW), in accordance with the woreda's Andilaye action planning document. Similarly, it was recommended that HEWs perform supportive supervision with each WDAL in her catchment area every month, in accordance with the HEW's Andilaye action planning document. When conducting supervisory visits, HEWs were trained to use the supportive supervision and on-the-job checklist. However, endline monitoring surveys conducted 2-3 months after the review meeting and refresher training of WDALs (round 2) indicated that few HEWs have received supportive supervision and on-the-job training from CHC HEW Supervisors and that few WDALs have received supportive supervision and on-the-job training from HEWs.				
**(7) Skills-based refresher training of community conversation facilitators**					
District-level maintenance activity		Bahir Dar Zuria	Fogera	Farta	Overall
Dose delivered	Proportion of study districts with activity implemented (February-April 2019)	1/1 (100%) [a]	1/1 (100%)	1/1 (100%)	3/3 (100%)

	Proportion of study districts with activity objectives completed as planned (according to activity checklist)	1/1 (100%)	1/1 (100%)	1/1 (100%)	3/3 (100%)
	Length of activity (one day training)	8 hours	8.5 hours	7.75 hours	8 hours
Participation	Total number of participants	34	25	46 [b]	105
	Proportion of study districts with Woreda Health Office representative AND Hygiene and Sanitation Officer in attendance	1/1 (100%)	0/1 (0%)	1/1 (100%)	2/3 (66%)
	Proportion of Andilaye community conversation facilitators in attendance	32/32 (100%)	24/24 (100%)	44/44 (100%)	100/100 (100%)
	Proportion of intervention kebeles with at least at least one facilitator per pair of facilitators trained on Andilaye community conversations	8/8 (100%)	6/6 (100%)	11/11 (100%)	25/25 (100%)
Does received	Proportion of intervention kebeles that identified implementation challenges and developed action plan for ways forward for the completion of group-level activities	8/8 (100%)	6/6 (100%)	11/11 (100%)	25/25 (100%)
Context	[a] Considerable delays were faced when trying to facilitate the Bahir Dar refresher training. While other woredas had trainings in February 2019, Bahir Dar Zuria's refresher training was delayed until early April 2019. These delays resulted from the woreda's prioritization in national health care campaigns in which many trained community conversation facilitators were involved (HEWs, kebele administrators, kebele managers, etc.). Accordingly, unlike in the original training of community conversation facilitators, all participants were trained together in Bahir Dar Zuria.<br>[b] Two training sessions were conducted in Farta given the high number of intervention kebeles and target participants.				

Supplemental Table 3c. Process data for key *Andilaye* behavior change activities

(1) Counseling visits and follow-up barrier planning counseling visits with caregivers					
Household-level catalyzing and maintenance activity		Bahir Dar Zuria	Fogera	Farta	Overall
**Dose delivered**	Proportion of intervention *kebeles* with activity implemented (February 2018-May 2019)	8/8 (100%)	6/6 (100%)	11/11 (100%)	25/25 (100%)
	Proportion of respondents from study-enrolled households[¥] in intervention *kebeles* reporting they received an *Andilaye* counseling visit from a WDAL [during endline household visits in March to May 2019]	126/220 (57%)	75/146 (51%)	190/299 (64%)	391/665 (59%)
	Proportion of respondents from study-enrolled households[¥] in intervention *kebeles* reporting they received more than one *Andilaye* counseling visit from a WDAL [during endline household visits in March to May 2019]	83/220 (38%)	54/146 (37%)	149/299 (50%)	286/665 (43%)
	Average number of *Andilaye* counseling visits reported of study-enrolled households[¥] reporting at least one *Andilaye* counseling visit [during endline household visits in March to May 2019]	298/126 (2.4)	189/75 (2.5)	559/190 (2.9)	1,046/391 (2.7) [a]
**Participation**	Proportion of study-enrolled households in intervention *kebeles* with an *Andilaye* goal card observed in the house [during endline household visits in March to May 2019]	142/241 (59%)	95/171 (56%)	246/331 (74%)	483/743 (65%) [b]
	Proportion of respondents from study-enrolled households[¥] in intervention *kebeles* that could identify the WDAL responsible for conducting their *Andilaye* counseling visits [during endline household visits in March to May 2019]	140/220 (64%)	88/146 (60%)	211/299 (71%)	439/665 (66%)
**Dose received**	Proportion of respondents from study-enrolled households in intervention *kebeles* with at least one *Andilaye* counseling visit reporting they set household goals or incremental improvements [during endline household visits in March to May 2019]	86/126 (68%)	57/75 (76%)	138/190 (73%)	281/391 (72%) [b]
	Proportion of respondents from study-enrolled households in intervention *kebeles* with at least one *Andilaye* counseling visit reporting they discussed barriers to the goals or incremental improvements [during endline household visits in March to May 2019]	75/126 (60%)	52/75 (70%)	131/190 (69%)	258/391 (66%) [b]
	Proportion of respondents from study-enrolled households in intervention *kebeles* with at least one *Andilaye* counseling visit reporting they discussed solutions to the barriers of the goals or incremental improvements [during endline household visits in March to May 2019]	72/126 (57%)	46/75 (61%)	124/190 (65%)	242/391 (62%) [b]
**Context**	[¥] Excluding study-enrolled households that were residents of caregivers who were trained as WDALs responsible for conducting the *Andilaye* counselling visits with caregivers (Bahir Dar Zuria, n=5; Fogera, n=15; Farta, n=22)				

	[a] Per protocol, WDALs were to act as the primary counselor and visit each household in her catchment area to conduct an *Andilaye* household counselling visit about once per month, with each visit lasting around 30 minutes. WDALs from all intervention *kebeles* implemented *Andilaye* counseling visits with caregivers. However, no *kebele* had WDALs conducting monthly counseling visits according to caregivers from study-enrolled households in intervention *kebeles* surveyed during endline. Of households reporting at least one visit (n=391), the average number of visits was 2-3 during the 14-15 months of implementation (i.e., since the initial trainings in January-February 2018). This suggests visits were likely only conducted following each round of WDAL training (n=3) for a majority of WDALs.<br>[b] All behavior change techniques designed into the *Andilaye* counseling visits with caregivers (e.g., inter-personal counseling on action planning, barrier identification and planning; goal setting, commitment, and self-regulation), were critical to the *Andilaye* intervention. However, of the households reporting at least one counseling visit (n=391), only 72% reported that they set household goals or incremental improvements, and two-thirds reported that they identified barriers, and their WDAL provided counseling on how to plan for, cope with, and overcome barriers – suggesting that some households receiving visits were not exposed to the intended intervention, as designed.				
**(2) Community mobilization and commitment event**					
Community-level catalyzing activity		Bahir Dar Zuria	Fogera	Farta	Overall
Dose delivered	Proportion of intervention *kebeles* with activity implemented (March-April 2018)	8/8 (100%)	6/6 (100%)	11/11 (100%)	25/25 (100%)
	Proportion of intervention *kebeles* with activity objectives completed as planned (according to activity checklist)	8/8 (100%)	6/6 (100%)	11/11 (100%)	25/25 (100%) [a]
	Average length of activity (partial day event)	2.33 hours	2 hours	2 hours	2 hours
Participation	Estimated average number of adult community members in attendance per event	355	298	279	309 [b]
	Proportion of respondents from study-enrolled households in intervention *kebeles* reporting being aware of the *Andilaye* community mobilization and commitment event [during quarterly monitoring visits in July/December 2018]	35/226 (15%)	47/172 (27%)	77/305 (25%)	159/703 (22%) [b]
	Proportion of respondents from study-enrolled households in intervention *kebeles* reporting they attended the *Andilaye* community mobilization and commitment event [during quarterly monitoring visits in July/December 2018]	29/226 (13%)	43/172 (25%)	56/305 (18%)	128/703 (18%) [b]
	Proportion of intervention *kebeles* with ALL coordinating committee members in attendance	3/8 (38%)	1/6 (17%)	5/11 (45%)	9/25 (36%) [c]
	Proportion of coordinating committee members in attendance	16/34 (47%)	15/27 (56%)	29/45 (64%)	60/106 (57%) [c]
	Proportion of intervention *kebeles* with ALL masters of ceremony in attendance	1/8 (13%)	3/6 (50%)	3/11 (27%)	9/25 (36%) [c]
	Proportion of masters of ceremony in attendance	14/22 (64%)	11/15 (73%)	20/31 (65%)	45/68 (66%) [c]
	Proportion of intervention *kebeles* with ALL HEWs in attendance	5/8 (63%)	3/6 (50%)	7/11 (64%)	15/25 (60%)
	Proportion of intervention *kebeles* with at least one HEW in attendance	7/8 (88%)	6/6 (100%)	11/11 (100%)	24/25 (96%) [d]
	Proportion of intervention *kebeles* with ALL *Andilaye* trained WDALs in attendance	1/8 (13%)	3/6 (50%)	5/11 (45%)	9/25 (36%)
	Proportion of *Andilaye* trained WDALs in attendance	39/73 (53%)	41/56 (73%)	84/110 (76%)	164/229 (72%)

Dose received	Proportion of intervention *kebeles* that determined practices no longer deemed to be acceptable by the community at the end of the event	8/8 (100%)	6/6 (100%)	10/11 (91%)	24/25 (96%)
	Proportion of intervention *kebeles* that determined improved behaviors at the end of the event	8/8 (100%)	6/6 (100%)	9/11 (82%)	23/25 (92%)
	Proportion of intervention *kebeles* that determined regulations for monitoring the by-laws at the end of the event	6/8 (75%)	6/6 (100%)	9/11 (82%)	21/25 (84%) [e]
Context	[a] The objectives were completed as planned, however, slight difference in the quality of event performance groups hired for skits and music during the events were noticed. Four performance groups were hired to perform in the 25 intervention *kebeles*. The two performance groups hired from Farta who performed in *kebeles* in Farta and Fogera performed better than the two performance groups who were hired from Bahir Dar Zuria. Since equal payment was decided for all performance groups hired, it was not possible to get best performance groups in Bahir Dar Zuria because of more opportunities for performance groups in Bahir Dar Zuria than in Farta (i.e., higher quality Bahir Dar Zuria performance groups were more expensive).<br>[b] Mobilizing community members to come to the event presented a challenge in participation. Mobilization and overall community planning of the event was tasked to the coordinating committee and masters of ceremony identified during the 'Whole System in the Room' activity. The payment (per diem) issue associated with the 'Whole System in the Room' might have impacted the motivation of some coordinating committee members and masters of ceremony to follow through with activities identified in the action plan.<br>[c] Many absent coordinating committee members and masters of ceremony were reportedly engaged in other government meetings during the day of the event. The Ethiopian-based study team was in attendance for all events and efforts by the team were made to assist coordinating committee members and masters of ceremony that were in attendance to mobilize and conduct the event.<br>[d] HEW turnover presented a challenge in HEW attendance in one intervention *kebele*.<br>[e] Setting community by-laws and determining regulations for monitoring the by-laws was a challenge in some *kebeles*. In few *kebeles*, HEWs expressed their concern on determining regulations considering the current political situation (i.e., recent declarations of states of emergency) and public protests in the region in the last few years. In one *kebele*, it was noted that practices no longer deemed acceptable by the community and practices that need to be improved were already determined in previous WASH meetings and the community leaders stated there is no need to do the same thing again; the community leaders agreed to write the already set by-laws on the banner later the day. In another *kebele*, the *kebele* administrator and manager were not willing to lead a discussion for developing by-laws and left the event before the pledge and commitment event. Two other *kebele*, agreed to determine regulations for monitoring the by-laws in the following weeks of the event.				
**(3) Community conversations and follow up community conversations**					
Group-level catalyzing and maintenance activity		Bahir Dar Zuria	Fogera	Farta	Overall
Dose delivered	Proportion of intervention *kebeles* with activity implemented (October 2018-May 2019)	6/8 (75%)	6/6 (100%)	11/11 (100%)	23/25 (92%) [a]
	Proportion of intervention *kebeles* with facilitators reporting they completed activity as designed (i.e., at least two groups of community members attending community conversation sessions for all three behavioral themes) [during endline reports in May 2019]	2/8 (25%)	5/6 (83%)	7/11 (64%)	14/25 (56%)
Participation	Proportion of respondents from study-enrolled households in intervention *kebeles* reporting that they have heard about the *Andilaye* community conversations [during endline household visits in March to May 2019]	63/225 (28%)	76/161 (47%)	190/321 (57%)	329/707 (46%)

	Proportion of respondents from study-enrolled households in intervention *kebeles* reporting they attended at least one *Andilaye* community conversations [during endline household visits in May 2019]	30/225 (13%)	48/161 (30%)	122/321 (38%)	200/707 (28%) [b]
Context	[a] Community conversation facilitators from two *kebeles* in Bahir Dar Zuria reported that they had not conducted community conversations within the 8 months since the initial training. Facilitators reported competing priorities that require the immediate action of facilitators. Overall, facilitators reported that punctuality and motivation of participants as a challenge in conducting community conversations. During refresher trainings (February-April 2019), many facilitators suggested addressing these issues by encouraging participation within the community by-laws as well as working with *kebele* officials to mobilize households.<br>[b] While the reported attendance of respondents is low, it should be noted that community conversations were designed to address a variety of participants beyond primary caregivers (such as, husbands/fathers, community or religious leaders/elders, youths/students, *kebele* officials/administrators, health development leaders, etc.).				
