# Supplementary material for: The impact of a demand-side sanitation and hygiene promotion intervention on sustained behavior change and health in Amhara, Ethiopia: A cluster-randomized trial
Source: PLOS Glob Public Health. 2022 Jan 7;2(1):e0000056. doi: 10.1371/journal.pgph.0000056 (PMC10021625; doi:10.1371/journal.pgph.0000056)
Supplement: S4 Table — (PDF) [file pgph.0000056.s005.pdf]

Supplemental Table 4. *Andilaye* Trial indicators used to assess WASH behavior

Indicators	Overall		Intervention		Control			
**Sanitation**								
**Household latrine coverage**	**N**	**%**	**N**	**%**	**N**	**%**	**PR (95% CI)[a]**	**PD (95% CI)[b]**
Households with at least one latrine	1472	61.6	743	61.2	729	62.0	0.99 (0.82, 1.21)	0.00 (-0.12, 0.12)
Households with improved latrine	1467	32.5	741	34.6	726	30.3	1.13 (0.81, 1.59)	0.04 (-0.07, 0.15)
Households with latrine with smooth and cleanable slab/floor	1471	14.8	743	16.3	728	13.3	1.19 (0.70, 2.03)	0.03 (-0.05, 0.11)
Households with fully constructed latrine	1471	30.9	742	33.0	729	28.7	1.15 (0.86, 1.54)	0.04 (-0.46, 0.13)
Percent of household latrines that were fully constructed	906	50.1	454	53.4	452	46.2	**1.18 (1.01, 1.38)**	**0.08 (0.01, 0.16)**
**Household latrine characteristics**								
Stagnant water present over the floor / latrine slab	906	17.0	455	16.3	451	17.7	0.92 (0.66, 1.28)	-0.01 (-0.07, 0.04)
Pan, slab, or floor was discolored (e.g., yellow, green)	904	59.7	454	58.4	450	61.1	0.97 (0.88, 1.08)	-0.02 (-0.08, 0.04)
Presence of flies in latrine	906	83.2	455	81.8	451	84.7	0.97 (0.89, 1.06)	-0.02 (-0.95,0.05)
Presence of drop hole cover in the latrine	906	14.1	455	18.2	451	10.0	**1.77 (1.19, 2.63)**	**0.08 (0.02, 0.14)**
Among those with a drop hole, a cover was situated over drop hole	130	67.7	85	70.6	45	62.2	1.09 (0.88, 1.36)	0.06 (-0.08, 0.20)
Presence of cleaning agents for washing latrine	906	5.7	455	8.4	451	3.1	2.46 (0.76, 7.96)	0.04 (-0.02, 0.11)
Presence of feces on floor / slab or other place in the latrine	906	50.6	455	50.1	451	51.0	1.02 (0.90, 1.15)	0.01 (-0.05, 0.07)
Evidence latrine is used for storage or other non-sanitation-related purpose	906	7.6	455	7.7	451	7.5	0.93 (0.49, 1.75)	-0.01 (-0.05, 0.04)
Presence of well-worn path to latrine	906	95.1	455	95.4	451	94.9	1.01 (0.97, 1.05)	0.00 (-0.03, 0.04)
Presence of fresh feces on / in the pit or pan	906	73.3	455	73.6	451	73.0	1.01 (0.90, 1.12)	0.00 (-0.07, 0.08)
Pit is full or close to being full	905	12.2	454	11.7	451	12.6	0.92 (0.57, 1.49)	-0.01 (-0.68, 0.05)
Presence of anal cleansing item inside or near latrine	906	40.3	455	39.1	451	41.5	0.93 (0.77, 1.12)	-0.03 (-1.02, 0.04)
Presence of odor from stool or urine in the latrine	906	84.0	455	81.8	451	86.3	0.96 (0.87, 1.04)	-0.04 (-0.11, 0.04)
Presence of leaves, spider webs, rubbish, other dirt in latrine	906	20.3	455	18.5	451	22.2	0.85 (0.60, 1.12)	-0.03 (-0.11, 0.04)
Wet latrine floor	906	43.8	455	40.7	451	47.0	0.85 (0.68, 1.07)	-0.07 (-0.16, 0.03)
Presence of water available near or inside latrine for hand washing	906	12.3	455	18.0	451	6.4	**2.28 (1.08, 4.81)**	0.09 (-0.01, 0.18)
Presence of cleansing agent near or inside latrine for hand washing	905	2.8	454	5.3	451	0.2	**21.67 (2.17, 215.98)**	0.07 (-0.04, 0.18)
Water available inside or near latrine for flushing or self-cleansing	906	4.5	455	6.6	451	2.4	**2.35 (1.05, 5.28)**	0.04 (-0.01, 0.08)
**Household latrine facility operation and maintenance**								
Latrine cleaned during the last seven days	904	23.2	454	25.6	450	20.9	1.02 (0.65, 1.60)	0.00 (-0.95, 1.03)
Household has added or improved anything on this latrine since its original construction	899	16.5	453	17.2	446	15.7	1.08 (0.71, 1.65)	0.01 (-0.06, 0.08)
Have ever fixed anything that became broken, damaged, our worn out on this latrine since its original construction	900	23.1	453	23.4	447	22.8	1.02 (0.76, 1.37)	0.00 (-0.06, 0.07)
Is your latrine facility working (operating) correctly now?	907	93.4	455	94.1	452	92.7	1.02 (0.98, 1.05)	0.01 (-0.02, 0.05)
Facility observed to require obvious repair	906	75.3	455	70.1	451	80.5	**0.88 (0.78, 0.99)**	**-0.10 (-0.19, -0.01)**
The latrine was observed to be serviceable	906	78.8	455	80.0	451	77.6	1.05 (0.93, 1.18)	0.04 (-0.05, 0.13)
Cleaning agents for washing latrine were observed inside or near the latrine	906	5.7	455	8.4	451	3.1	2.46 (0.76, 7.96)	0.04 (-0.03, 0.11)

Presence of feces on floor, slab, or other place in the latrine aside from the pit	906	50.1	455	50.1	451	51.0	1.02 (0.90, 1.15)	0.01 (-0.05, 0.07)
**Latrine utilization**	**N**	**mean (SE)**	**N**	**mean (SE)**	**N**	**mean (SE)**	**-**	**difference (95% CI)[e]**
Given you have a latrine, number of households that used this household latrine in last 7 days	905	1.63 (0.09)	454	1.54 (0.09)	451	1.72 (0.14)	-	-0.11 (-0.42, 0.19)
Given household has a latrine, number of people who used this latrine from another household during last 7 days, not including your household members	902	0.91 (0.14)	454	0.70 (0.16)	448	1.11 (0.22)	-	-0.40 (-0.85, 0.05)
	**N**	**%**	**N**	**%**	**N**	**%**	**PR (95% CI)[a]**	**PD (95% CI)[b]**
Given you have a latrine, household members have used this latrine for 3 or more days during the last 7 days	905	90.8	455	92.1	450	89.6	1.04 (0.99, 1.10)	0.04 (-0.13, 0.09)
Respondent defecated in any latrine during last 2 days	1469	45.5	740	45.8	729	45.3	1.01 (0.79, 1.29)	0.00 (-0.11, 0.12)
Respondent always exclusively used a latrine for defecation during last 7 days	1472	46.4	743	53.2	729	54.1	0.99 (0.79, 1.24)	-0.00 (-0.12, 0.12)
Respondent's primary place of defecation changes over the course of the year	1470	27.3	742	28.8	728	25.7	1.16 (0.90, 1.50)	0.04 (-0.03, 0.11)
Head of household defecated in any latrine during last 2 days	900	57.7	486	57.6	414	57.7	1.01 (0.83, 1.23)	0.01 (-0.11, 0.12)
Head of household always exclusively used a latrine for defecation during last 7 days	1002	34.8	529	36.5	473	33.0	1.07 (0.79, 1.47)	0.03 (-0.09, 0.15)
Head of household's primary place of defecation changes over the course of the year	1205	25.9	624	25.5	581	26.3	0.96 (0.71, 1.30)	-0.01 (-0.09, 0.07)
Ages 4-17 defecated in any latrine during last 2 days	2991	52.1	1538	52.7	1453	51.2	1.01 (0.83, 1.20)	0.00 (-0.10, 0.11)
Ages 4-17 always exclusively used a latrine for defecation during last 7 days	2842	38.9	1447	42.6	1385	35.0	1.15 (0.89, 1.50)	0.06 (-0.05, 0.16)
Ages 4-17 primary place of defecation changes over the course of the year	3532	23.5	1778	24.5	1754	22.4	1.08 (0.83, 1.41)	0.02 (-0.05, 0.08)
Safely disposed of child feces	777	38.9	401	36.7	376	41.2	0.96 (0.69, 1.32)	-0.02 (-0.15, 0.11)
**Open defecation practices**								
Respondent's primary place of defecation was OD during last 2 days	1472	39.5	743	40.2	729	38.8	1.05 (0.76, 1.45)	0.02 (-0.11, 0.15)
Respondent openly defecated during last 2 days	1472	44.9	743	45.2	729	44.9	1.03 (0.78, 1.36)	0.01 (-0.11, 0.14)
Respondent openly defecated in or near surface water	660	10.9	335	10.2	325	11.7	0.93 (0.50, 1.72)	-0.01 (-0.08, 0.06)
Respondent urinated in/or near surface water	1466	7.2	739	6.5	727	8.0	0.82 (0.46, 1.48)	-0.01 (-0.06, 0.03)
Head of household's primary place of defecation was OD during last 2 days	1146	40.3	600	39.7	546	41.0	0.95 (0.68, 1.31)	-0.02 (-0.15, 0.11)
Head of household openly defecated during last 2 days	992	64.7	527	62.6	465	67.1	0.98 (0.80, 1.21)	-0.01 (-0.14, 0.12)
Head of household openly defecated in or near surface water during last 2 days	371	12.4	199	11.6	172	13.3	1.07 (0.48, 2.40)	0.01 (-0.09, 0.10)
Head of household urinated in/near surface water	704	7.7	387	6.7	317	8.8	0.82 (0.35, 1.94)	-0.01 (-0.08, 0.05)
Ages 4-17 primary place of defecation was OD during last 2 days	3494	44.3	1757	42.9	1737	45.7	0.97 (0.72, 1.29)	-0.01 (-0.14, 0.11)
Ages 4-17 openly defecated during last 2 days	2921	58.8	1489	55.2	1432	62.6	0.95 (0.77, 1.16)	-0.03 (-0.15, 0.09)
Ages 4-17 openly defecated in or near surface water during last 2 days	1326	18.4	629	14.5	697	22.0	0.73 (0.39, 1.37)	-0.05 (-0.17, 0.06)

Ages 4-17 urinated in/near surface water	2480	11.7	1273	9.0	1207	14.5	0.66 (0.35, 1.24)	-0.05 (-0.12, 0.03)
Evidence of open defecation (i.e., human feces) in/near household compound	1472	45.2	743	43.5	729	47.1	0.95 (0.78, 1.15)	-0.03 (-0.11, 0.06)
**Personal hygiene**								
**Washing station coverage**								
Reported HH hand or facewashing station(s)	1472	98.0	743	98.3	729	97.7	1.01 (0.99, 1.02)	0.01 (-0.01, 0.02)
Observed HH hand or facewashing station(s) with water	1472	19.9	743	19.5	729	20.3	0.96 (0.72, 1.26)	-0.01 (-0.06, 0.05)
Observed HH hand or facewashing station(s) with soap (%)	1472	2.2	743	2.6	729	1.9	1.34 (0.44, 4.13)	0.01 (-0.02, 0.04)
**Handwashing practices**								
Yesterday, the index child's hands were washed	1428	98.5	724	99.0	704	97.9	**1.01 (1.00, 1.02)**	**0.01 (0.00, 0.02)**
The last time the index child's hands were washed, soap/ash/or soapy water used	1398	44.8	714	46.5	684	43.0	1.08 (0.89, 1.30)	0.03 (-0.05, 0.12)
The last time the index child defecated, he/she cleaned hands with water and soap, soapy water, or ash	1410	41.8	713	43.9	697	39.6	1.12 (0.92, 1.35)	0.05 (-0.04, 0.13)
Yesterday, the respondent washed his/her hands with water	1469	98.0	742	98.3	727	97.8	1.01 (0.99, 1.02)	0.01 (-0.01, 0.02)
The last time the respondent washed he/she used soap/ash/soapy water	1468	44.0	740	46.0	728	42.0	1.09 (0.91, 1.31)	0.04 (-0.04, 0.12)
The last time the respondent defecated, he/she cleaned hands with water and soap	1463	49.0	738	51.9	725	46.1	1.13 (0.94, 1.35)	0.06 (-0.03, 0.15)
The last time the respondent prepared food, he/she cleaned hands with water and soap before beginning food preparations	1403	51.0	700	53.6	703	48.5	1.11 (0.95, 1.29)	0.05 (-0.03, 0.13)
**Hand cleanliness**								
Respondent’s finger nails clean on left hand	1472	73.6	743	74.6	729	72.7	1.03 (0.96, 1.11)	0.02 (-0.03, 0.08)
Respondent’s finger nail clean on right hand	1472	73.4	743	73.6	729	73.1	1.01 (0.94, 1.09)	0.01 (-0.04, 0.65)
Respondent’s finger pads clean on left hand	1472	62.1	743	63.3	729	60.9	1.05 (0.95, 1.15)	0.03 (-0.03, 0.09)
Respondent’s Finger pads clean on right hand	1472	62.2	743	62.7	729	61.7	1.03 (0.93, 1.13)	0.02 (-0.04, 0.08)
Respondent’s palm clean on left hand	1472	57.5	743	59.0	729	56.1	1.05 (0.94, 1.18)	0.03 (-0.04, 0.09)
Respondent’s palm clean on right hand	1472	57.9	743	59.1	729	56.6	1.05 (0.94, 1.17)	0.03 (-0.04, 0.09)
Index child’s finger nails clean on left hand	1024	84.1	502	83.3	522	84.9	0.96 (0.91, 1.02)	-0.03 (-0.08, 0.14)
Index child’s finger nail clean on right hand	1024	83.9	502	83.1	522	84.7	0.96 (0.90, 1.02)	-0.03 (-0.08, 0.02)
Index child’s finger pads clean on left hand	1024	80.6	502	79.9	522	81.2	0.98 (0.91, 1.05)	-0.02 (-0.07, 0.03)
Index child’s finger pads clean on right hand	1024	80.4	502	79.7	522	81.0	0.98 (0.91, 1.05)	-0.02 (-0.07, 0.03)
Index child’s palm clean on left hand	1024	78.9	502	78.9	522	78.9	0.99 (0.93, 1.05)	-0.01 (-0.06, 0.04)
Index child’s palm clean on right hand	1024	78.6	502	78.5	522	78.7	0.98 (0.92, 1.05)	-0.01 (-0.06, 0.04)
**Facewashing practices**								
Yesterday, the index child's face was cleaned	1433	98.1	723	97.9	710	98.3	0.99 (0.98, 1.01)	-0.01 (-0.02, 0.01)
Yesterday, after index child’s face was washed it was it wiped dry with a cloth	1325	37.1	665	38.1	660	36.2	1.04 (0.90, 1.21)	0.02 (-0.04, 0.07)
Yesterday, the respondent cleaned his/her face	1470	98.0	742	97.6	728	98.4	0.99 (0.97, 1.01)	-0.01 (-0.03, 0.01)
Yesterday, the respondent wiped his/her face dry with a cloth such as a towel or apron	1393	39.1	702	40.9	691	37.3	1.09 (0.09, 1.27)	0.03 (-0.03, 0.10)
**Face cleanliness**								

Ocular discharge is present (all children ages 1-9 years)	1696	28.7	822	26.9	874	30.4	0.88 (0.68, 1.15)	-0.04 (-0.11, 0.04)
Wet nasal discharge is present (all children ages 1-9 years)	1696	38.2	822	37.0	874	39.4	0.94 (0.78, 1.13)	-0.02 (-0.09, 0.05)
Dry nasal discharge is present (all children ages 1-9 years)	1696	44.0	822	42.7	874	45.2	0.97 (0.81, 1.16)	-0.02 (-0.10, 0.06)
Dirt/dust/other debris is present (all children ages 1-9 years)	1696	50.0	822	50.5	874	49.5	1.03 (0.89, 1.20)	0.02 (-0.06, 0.09)
Ocular discharge is present (index child)	1024	29.8	502	27.5	522	32.0	0.88 (0.68, 1.15)	-0.04 (-0.11, 0.04)
Wet nasal discharge is present (index child)	1024	39.8	502	37.7	522	42.0	0.94 (0.78, 1.13)	-0.02 (-0.09, 0.05)
Dry nasal discharge is present (index child)	1024	45.6	502	43.2	522	47.9	0.97 (0.81, 1.16)	-0.02 (-0.05, 0.06)
Dirt/dust/other debris is present (index child)	1024	53.0	502	52.2	522	52.9	1.03 (0.89, 1.20)	0.02 (-0.06, 0.09)
	**N**	**mean (SE)**	**N**	**mean (SE)**	**N**	**mean (SE)**		**difference (95% CI)[e]**
Number of times a fly land on the index child's face during a 1 minute observation	1024	3.3 (0.21)	502	3.2 (0.33)	522	3.4 (0.26)	-	-0.15 (-0.90, 0.60)
**Household environmental sanitation**								
**Animal husbandry and hygiene practices**	**N**	**%**	**N**	**%**	**N**	**%**	**PR (95% CI)[a]**	**PD (95% CI)[b]**
Respondent has animal herding or other animal husbandry responsibilities	1472	87.4	743	88.3	729	86.4	1.02 (0.96, 1.09)	0.02 (-0.04, 0.07)
Head of household has animal herding or other animal husbandry responsibilities	1243	91.4	639	92.5	604	90.2	1.05 (0.99, 1.11)	0.04 (-0.01, 0.09)
Observed animal feces present in the compound	1472	82.3	743	82.2	729	82.4	1.01 (0.92, 1.11)	0.01 (-0.07, 0.08)
Animal feces/waste not left out in open in compound	1472	53.8	743	56.4	729	51.2	1.10 (0.95, 1.28)	0.05 (-0.03, 0.13)
**Solid waste management**								
Solid waste was not observed to have been left out in the open	1472	31.1	743	34.6	729	27.6	1.26 (0.93, 1.69)	0.07 (-0.02, 0.17)
