# Supplementary material for: The impact of a demand-side sanitation and hygiene promotion intervention on sustained behavior change and health in Amhara, Ethiopia: A cluster-randomized trial
Source: PLOS Glob Public Health. 2022 Jan 7;2(1):e0000056. doi: 10.1371/journal.pgph.0000056 (PMC10021625; doi:10.1371/journal.pgph.0000056)
Supplement: Attachment — Submitted filename: Response to reviewers 07Oct2021.docx [file pgph.0000056.s006.docx]

We are pleased to submit a revision of our manuscript. We below we provide line-by-line responses to the editor and reviewers.

Editorial comments:

1. Review and correct the sentence on lines 112-3, which seems to be missing a clause.

>> Added

2. Ensure that Table 1 is located in the results section, rather than the methods section.

>> Table 1 has been moved

Reviewer suggestions:

Reviewer #1: You have clearly conducted a scientifically sound analysis to investigate a contextually significant issue.

>> We thank the reviewer for their constructive suggestions

I have a few minor suggestions for clarification. What is your baseline, and what distinguishes Andilaye from Community-Led Total Sanitation (CLTS), Participatory Hygiene and Sanitation Transformation (PHAST), and Ethiopian Health Extension Program at the start of its development?

>> Regarding the question on baseline data: Kebele and household enrollment took place during baseline data collection (March to April 2017) prior to the implementation of Andilaye activities (see Figure 2 for details on the study timeline). As mentioned in the methods: “Households with at least one child under five years were randomly selected using systematic random sampling at baseline, and were followed for each round of data collection.”

Regarding the question about what distinguishes Andilaye from other demand-side approaches: Within the last paragraphs of our introduction, we highlight documented limitations of CLTHS. Most importantly, “The use of negative affective motivators employed by CLTS(H) may not be culturally appropriate or the most effective drivers of sanitation and hygiene behavior change [39], and may erode mental well-being. Together, the focus on negative affective motivators, poor facilitation of initial triggering, and a lack of follow-up, has left many communities with negative impressions of CLTSH initiatives.” We highlight how “The [Andilaye] intervention takes a positive, encouragement approach to behavior change by promoting incremental improvements in behavior and incorporating behavioral maintenance strategies to foster sustained behavior change.” In our methods, we note that “Baseline WASH and demographic statistics, along with the fact that 39 of 50 kebele clusters randomly selected for inclusion in the Andilaye Trial had been triggered with CLTSH, and certified ODF, provided strong evidence that behavioral slippage was, indeed, an issue that needed to be addressed in Amhara and perhaps elsewhere in Ethiopia.” We include considerable documentation about the intervention approach, including household goal cards and a focus on moving up the sanitation ladder and targeting more than open defecation that distinguishes this approach. PHAST has a well-documented methodology that differs considerably from Andilaye.

How to deal with the contamination of information on the during Andilaye intervention.

>> We have provided further information in the methods section from our referenced protocol to describe study design elements to address spill over: “We utilized a ‘fried egg’ [50] approach to purposively select one to two gotts that were either situated in or near the center of the kebele (if there were centric gotts) or were not adjacent to any other study kebele (in the event there are no centric gotts). This approach minimized spill-over of intervention effects and other externalities associated with the research between intervention and control clusters, especially those adjacent to each other.”

Reviewer #2: The authors do an excellent job motivating the study. I appreciated that this was an effectiveness study, contrasted very clearly in the discussion with an efficacy study to point out the challenges of scaling such interventions. I suggest some areas for improvement, but I felt that the paper overall was well written and concisely described the study and results. There are a couple instances where some field-specific terms could be better explained to improve accessibility for broader PLOS GPH readers. My main comment is for the discussion section, where I do think the low household exposure to activities should be further discussed. Overall, I commend the authors for this important study and clear reporting of results and would look forward to reading this in its final form in PLOS GPH.

>> We thank the reviewer for their comments and suggestions. We have reviewed the discussion and provided additional clarity related to household exposure to activities as suggested above and below.

Specific areas for improvement:

I really do think the process evaluation results and particularly the low household exposure to the intervention should be included even more in the discussion. Are the null results surprising given the cascade of low implementation fidelity and even lower household exposure? Even if there is high dose delivered/received at levels above the household, outcomes are measured at the household level.

>> We have discussed this suggestion extensively among co-authors, specifically providing sufficient information on the fidelity, but without overemphasizing or overstepping our available data. We believe this point is sufficiently addressed: “The *Andilaye* intervention generally did not improve WASH conditions or outcomes. Without sustained changes to these WASH conditions and behaviors, changes in well-being were not likely, and indeed, were not detected. ... We believe that poor fidelity of intervention delivery played a considerable role in uptake of the intervention, pointing to challenges in delivering demand-side sanitation and hygiene interventions at scale and through existing community-based models.”

There was a high dose delivered, as defined by delivery of trainings, but low dose received at the household level by the WDALs/HEWs. We believe that our results and discussion adequately address this issue.

I do very much appreciate the inclusion in the discussion of the problematic reliance on unpaid women’s labor (women as WDALs), but I would have liked to see additional discussion of the women respondents (especially as women’s mental well-being is addressed in detail in the intro as motivation for the study). The fact that there was an impact on reported sanitation-related social support, even with this low exposure, is promising. (And I’m noting also the non-statistically-significant, but still trending toward improved, mental health outcomes shown in Table 2.)
>> We have added text in the results to highlight trends in mental-health measures and re-address this in the discussion “we saw some, non-statistical, preventive trends across all mental health measure which were primarily among women (90% of respondents). Our impact on sanitation social support is indicative of the underlying philosophy of the Andilaye intervention, which was designed contrary to the “shame” drivers of more traditional CLTSH [28,65]. These findings are encouraging despite the low fidelity of the intervention delivery at household -level, suggesting improved fidelity may result in evidence of impact.”

In the discussion, we added specific gender-related text related to mental well being: “In rural India, women’s experiences of sanitation, as measured by a validated sanitation insecurity measure, were associated with well-being, anxiety, depression, and distress, even when women had access to a facility. Similarly, in urban Mozambique, latrine location and neighborhood violence were important determinants of safety perceptions and corresponding psychosocial stress. These findings highlight the need for interventions to consider the experience of sanitation beyond access to a facility alone.”

Minor issues

o Missing space in abstract

>> We didn’t find this space

o Harmonize format of reporting PRs in abstract with colon or equal sign or space

>> done

o Some very minor copy-editing errors throughout

>> we have reviewed the manuscript for copy editing

Table 1:

o I strongly suggest reducing the number of significant figures for the PDs to two decimal places

>> We have made that change.

o It would be helpful to include the total sample size (N) for each of the intervention and control groups at the top of the column, in addition to the number with each outcome (n) that is currently included

>> The totasl are in the column already. We have clarified by changing the header label

o Line 155: Suggest removing “clusters” and just referring to kebeles as the randomization units (“clusters” made me wonder if the unit was multiple kebeles)

>> We agree that the use of “Kebele clusters” could be interpreted as multiple kebeles per cluster. We have revised text within our methods to help clarify: “We employed a structured sampling strategy to randomly select 50 eligible clusters within our sampling frame. Clusters were defined as rural or peri-urban sub-districts or kebeles — the smallest government administrative unit in Ethiopia — that were accessible throughout the course of the year. Of the 50 kebeles enrolled into the study, 22 were selected from Farta, 12 from Fogera, and 16 from Bahir Dar Zuria.”

o Line 157: It is not totally clear how topographical conditions are related to the intervention, though I can speculate. I suggest editing to make that reasoning and link more clear.

>> We have provided context for this within study strengths of our discussion: “While CRTs tend to emphasize internal validity, we made considerable effort to enhance external validity. Our study was spread over three woredas in two zones, yielding a heterogeneous mix of contexts and topographical conditions - which serve as a proxy for factors such as soil type, access to markets, and flooding risk - and support external validity of the findings.”

o Line 188 onward: It’s not clear to me from the text how the intervention activities are delivered at the district level, if the randomization occurred at the kebele level (so there are both intervention and control groups within the district). I suggest clarifying a bit.

>> We have made the following edit: “The *Andilaye* intervention commenced with district-level capacity building activities, such as action planning to orient key government stakeholders and training of trainers who would facilitate intervention activities in *kebeles* allocated to receive the *Andilaye* intervention.”

o Line 193: Could you add some explanation of the ‘Whole System in the Room’ activity as well as of the cross-fertilization visits? The former comes up multiple times throughout the paper but is not clearly described.

>> We have made the following edit: Community-level activities began with a ‘Whole System in the Room’ [51] to engage key community stakeholders from selected gotts and kebeles, orient them to the Andilaye intervention, and facilitate their involvement in intervention action planning. We would also like to note that we provide Supplemental figure 1 - a diagram summarizing the Andilaye intervention and Supplemental table 1 - a summary of the Andilaye intervention activities and dates of delivery – including the aim and level for each activity.

o General note in intervention explanation – “district” and “household” seem well defined, but “group” and “community” are not. I think it would be helpful to explain a bit what is meant by those two levels.

>> We have made edits to the description of the Andilaye intervention to provide a narrative of Supplemental table 1, including clarifications of group and community activities.

o Line 216 and 268: “Ethiopia-based”

>> changed.

o Line 219: “standard of care”

>> changed.

o Line 220: Are these equivalence tests described anywhere? Looking at Figure 1, I agree that 19/25 and 20/25 are balanced arms, but I’m not sure that should be described as an equivalence analysis.

>>We agree and have changed the wording, especially because statistical tests are not appropriate for showing balance between arms. We have edited to say, “There were no meaningful differences in the number of previously CLTSH-triggered and ODF certified kebeles, between study arms.”

o Line 221: “Baseline statistics showing X, along with…” (suggest expanding for clarity)

>>” Baseline WASH and demographic statistics”

o Line 249: For diarrhea period prevalence, can you specify (e.g., 7-day and 2-day) recall period?

>> Added

o Line 284: Does this percent range include other disorders as well? Or specifically anxiety and depression? If the latter, please delete “of common mental disorders”

>> Changed

o Under analytical methods, please include which software/estimation commands that were used to estimate marginal effects

>> We have changed the text to read “using the post-estimation margins command~~s~~ to estimate the average marginal effects”

o Line 307: “accounted”

>> Changed

o Line 328: “…and well exceeded our minimum sample size requirement…”

>> Changed

o Lin 331: suggest replacing “by design” with “who were typically the primary caretakers”, or something similar

>> Changed

o Overall, I think it’s helpful for the reader when they are provided some quantitative metric alongside general descriptions of data such as “conditions were often poor” (e.g., line 380), especially if the evidence supporting this will be in SI rather than main text. For example, in this line, you could move the “e.g., presence of feces” to later in the sentence and also include the accompanying proportions.

>> We cite the table and it is easier, in our opinion, to digest the specifics in the table. Given that there are a great number of indicators, we would prefer to keep secondary data to the table and avoid cherry-picking findings to highlight.

oTable 2 & Table 3:

oIt seems like the differences should use fewer significant figures (the scores themselves only go to two decimal places)

>> Changed. But Josh, note one figure for your assistance.

o Table 3 caption – delete last “scale”

>> Changed.

o Line 450: just confirming all of the reported PRs are adjusted

>> We did not include the adjusted PRs. This was a randomized trial with generally good balance in covariates between the intervention and control arms at baseline. As such, we did not control for additional confounders. We only "accounted for the stratified design through the inclusion of the woreda indicator variable, and incorporated generalized estimating equations with robust standard errors to account for the clustering of observations within kebeles.”

o Line 513-517: Thank you for including this very important point!

>> most welcome

o Line 541: Please add a brief explanation of the “fried egg” approach.

>> See above response. We have provided edits to the methods section so describe the application of the “fried egg” approach to minimize spill over

o Line 562-564: Great takeaway and so important

>> thanks

o I think I am missing the full Figure 3 – unsure what the x-axis labels are

>> This has been fixed

o Figure 1: What’s a gott?

>> We have added a description of *gott* in the text directly above the reference to Figure 1.

o SI tables: Check significant figures

>> Done

o What is the WDAL exactly? This is unclear from the main text, but would be helpful to clarify, especially because the point about increasing responsibilities asked of WDALs is brought up as a problem.

>> We have moved the presentation of Supplemental 2 (which describes an alignment of relevant roles and responsibilities of the HEP and Andilaye Trial) to be at the beginning of the description of Andilaye intervention activities. We have also provided text to further clarify WDALs.
