# Supplementary material for: The impact of a demand-side sanitation and hygiene promotion intervention on sustained behavior change and health in Amhara, Ethiopia: A cluster-randomized trial
Source: PLOS Glob Public Health. 2022 Jan 7;2(1):e0000056. doi: 10.1371/journal.pgph.0000056 (PMC10021625; doi:10.1371/journal.pgph.0000056)
Supplement: Attachment — Submitted filename: Response to reviewers 23Nov2021.docx [file pgph.0000056.s007.docx]

We thank the reviewers for their comments. We have responded to each comment below.

Reviewer 2

I have 3 minor copy-editing suggestions (line numbers in the track-changed doc) in the edited lines:
line 216 - "dialogue"

- “dialog” is the American English spelling so we have retained it for consistency.

line 578 - remove comma

- Comma has been removed

line 584 - "non-statistically significant"

- We have made the change
